# GPS/MEMS INS Data Fusion and Map Matching in Urban Areas

**DOI:** 10.3390/s130911280

**Published:** 2013-08-23

**Authors:** Hone-Jay Chu, Guang-Je Tsai, Kai-Wei Chiang, Thanh-Trung Duong

**Affiliations:** Department of Geomatics, National Cheng Kung University, No.1, University Road, Tainan 701, Taiwan; E-Mails: honejaychu@gmail.com (H.-J.C.); a7760968@hotmail.com (G.-J.T.); kwchiang@mail.ncku.edu.tw (K.-W.C.)

**Keywords:** map-matching, GPS, MEMS IMU, Kalman filter

## Abstract

This paper presents an evaluation of the map-matching scheme of an integrated GPS/INS system in urban areas. Data fusion using a Kalman filter and map matching are effective approaches to improve the performance of navigation system applications based on GPS/MEMS IMUs. The study considers the curve-to-curve matching algorithm after Kalman filtering to correct mismatch and eliminate redundancy. By applying data fusion and map matching, the study easily accomplished mapping of a GPS/INS trajectory onto the road network. The results demonstrate the effectiveness of the algorithms in controlling the INS drift error and indicate the potential of low-cost MEMS IMUs in navigation applications.

## 1. Introduction

A critical component of a navigation system is the Global Positioning System (GPS). However, outages and multi-path phenomenon of GPS signals frequently occur in urban areas. Another navigation system, the inertial navigation system (INS), is an interesting complementary navigation system [1]. GPS has long-term stability with homogeneous accuracy, while the short-term stability with high navigation accuracy of the INS is excellent, but stand-alone INS positioning accuracy deteriorates rapidly with time. Thus, the integration of GPS and INS provides a high data rate of complete navigation solutions (*i.e.*, position, velocity, and attitude) with superior accuracy. Advances in Micro-Electro-Mechanical System (MEMS) inertial sensor technology have rendered the integrated GPS and INS a low-cost option for positioning and navigation. In data fusion, the Kalman Filter (KF) suffers from divergence in the approximations of the linearization process during GPS outages. Inertial sensor biases are not well estimated leading to residual drifts, especially when using MEMS-based inertial measurement units (IMUs) [2,3]. Previous studies applied KF to combine GPS and IMU data in vehicle and aircraft navigation [4,5]. Bevly [4] demonstrated the ability of a low-cost GPS receiver to reduce errors inherent in low-cost ground vehicle IMUs. Wendel *et al.* [5] addressed the development of an integrated navigation system based on MEMS IMU and GPS for an unmanned aerial vehicle (UAV).

To obtain a robust navigation system, mapping the position onto a spatial road map is necessary [6]. This spatial mismatch is particularly severe at junctions and built-up areas with complex routes. Quddus *et al.* [7] reviewed the literature on map matching and listed the advantages and drawbacks of the various algorithms. The commonly used methods are point-to-curve matching and curve-to-curve matching. The point-to-curve matching algorithm attempts to identify the curve (link) that is closest to a point. The approach uses a local or incremental algorithm that maps current positions onto a road line on a map. Cossaboom *et al.* [8] used KF to integrate GPS and INS in a loosely coupled scheme to enhance navigational solution while further improvement is achieved by the map matching. However, the approach only considers the current position and the matching accuracy is generally low. A more novel approach is curve-to-curve matching, which considers the estimated location as a curve consisting of a set of points. This algorithm achieves better accuracy based on certain distance measures and aligns an entire trajectory with a road map.

The main purpose of this study is to conduct KF and map matching by integrating GPS and INS data to identify the right trajectory and robust navigation, and to improve the matching navigation accuracy. In addition, this study compares the point-to-curve and curve-to-curve matching approaches in the process of using a road network map. Result shows that the curve-to-curve matching approach is more effective in navigation applications.

## 2. Methods

### 2.1. Data Fusion Algorithm

KF is used for data fusion in this study. KF addresses the general problem to estimate the state *x* of a discrete-time controlled process that is governed by the state equation as follows:

(1) $$x_{k}=\Phi_{k-1;k}x_{k-1}+w_{k}$$

Where $x_{k}={\left[\delta R\;\delta V\;\delta \psi \;b_{a}\;b_{g}\;s_{a}\;s_{g}\right]}_{21\times 1}^{T}$ is the state vector at time *k* including position error, velocity error, attitude error, accelerometer bias, gyroscope bias, accelerometer scale factor, and gyroscope scale factor, respectively, Ф*_k_*_−1;_*_k_* is the state transition matrix from time *k*−*1* to *k*, and *w_k_* is the system noise.

The measurement model is:

(2) $$z_{k}=Hx_{k}+{ɛ}_{k}$$

Since the aiding measurements from GPS are position and velocity, Equation (2) can be rewritten as:

(3) $$z_{k}=\left[\begin{array}{l} R_{\text{INS}}^{e}-R_{\text{GPS}}^{e}\\ V_{\text{INS}}^{e}-V_{\text{GPS}}^{e} \end{array}\right]=\left[\begin{array}{l} H_{R}\;0\\ 0\;H_{V} \end{array}\right]\;\left[\begin{array}{l} \delta R^{e}\\ \delta V^{e} \end{array}\right]+\left[\begin{array}{l} {ɛ}_{R}\\ {ɛ}_{V} \end{array}\right]$$

where $H_{R}=H_{V}=\left[\begin{array}{ccc} 1 & 0 & 0\\ 0 & 1 & 0\\ 0 & 0 & 1 \end{array}\right]$ are mapping matrices, *δR^e^* = (*δx^e^*, *δy^e^*, *δz^e^*)*^T^* is the position error vector; $\delta V^{e}={\left(\delta V_{x}^{e},\delta V_{y}^{e},\delta V_{z}^{e}\right)}^{T}$ is the velocity error vector expressed in the Earth-centered Earth-fixed frame (ECEF or e-frame); *ε_R_* and *ε_V_* are the position and velocity noise, respectively. $R_{\text{INS}}^{e}$ and $R_{\text{GPS}}^{e}$ are the positions in the e-frame obtained by INS and GPS, respectively. ${\text{V}}_{\text{INS}}^{e}$ and ${\text{V}}_{\text{GPS}}^{e}$ are the velocities in the e-frame obtained by INS and GPS, respectively.

In a KF-based system, the system noise (*w_k_*) and the measurement noise (*ε_k_*) are assumed to be independent normal distributions with zero mean and variances (*Q_k_* and *R_k_*), respectively:

(4) $$w_{k}\sim N(0,Q_{k}),\;{ɛ}_{k}\sim N(0,R_{k})$$

In fact, the system noise and measurement noise are not usually correctly estimated but can be modeled by the representation of a system noise model and measurement noise model. In practice, *Q_k_* is modeled based on sensor calibration and *R_k_* is modeled based on the GPS position and velocity uncertainty.

Now, let's define ${\hat{x}}_{k}^{-}$ and ${\text{P}}_{\text{k}}^{-}$ as the *a priori* state and associated variance matrix estimated at time *k* given the knowledge of the process prior to time *k*−*1*, where:

(5) $${\hat{\text{x}}}_{\text{k}}^{-}=\phi_{\text{k}-1;\text{k}}{\hat{\text{x}}}_{\text{k}-1}$$

(6) $${\text{P}}_{\text{k}}^{-}=\phi_{\text{k}-1;\text{k}}{\text{P}}_{\text{k}-1}\phi_{\text{k}-1;\text{k}}^{\text{T}}+{\text{Q}}_{\text{k}}$$

*x̂_k_* is the *a posteriori* state estimate at time *k* given the measurement *z_k_*. *A priori* and *a posteriori* estimate errors and associated covariance matrices are:

(7) $$e_{k}^{-}=x_{k}-{\hat{x}}_{k}^{-},\;P_{k}^{-}=E[e_{k}^{-}e_{k}^{-T}]$$

(8) $$e_{k}=x_{k}-{\hat{x}}_{k},\;P_{k}=E[e_{k}e_{k}^{T}]$$

The linear combination between the *a posteriori* state estimate *x̂_k_*, *a priori* state estimate ${\hat{x}}_{k}^{-}$, and a weighted difference between actual measurement *z_k_* and measurement prediction $H{\hat{x}}_{k}^{-}$ is:

(9) $${\hat{x}}_{k}={\hat{x}}_{k}^{-}+K(z_{k}-H{\hat{x}}_{k}^{-})$$

The associated covariance matrix P_k_ is determined as:

(10) $${\text{P}}_{\text{k}}={\text{P}}_{\text{k}}^{-}-{\text{K}}_{\text{k}}{\text{H}}_{\text{k}}{\text{P}}_{\text{k}}^{-}$$

where the equation $\left(z_{k}-H{\hat{x}}_{k}^{-}\right)$ is called measurement innovation or the residual. *K* is called as Kalman gain matrix and can be shown by:

(11) $$K_{k}=P_{k}^{-}H^{T}{\left(HP_{k}^{-}H^{T}+R_{k}\right)}^{-1}$$

The KF estimates a process by using a form of feedback control—the filter estimates the process state at some time and then obtains feedback in the form of measurements. As such, the KF updates in two steps each stage: time update and measurement update. The time update estimates the current state and error covariance for the next time step. The measurement update incorporates a new measurement into the *a priori* estimate to obtain an improved *a posteriori* estimate.

### 2.2. Map-Matching Algorithm

**Figure 1.**
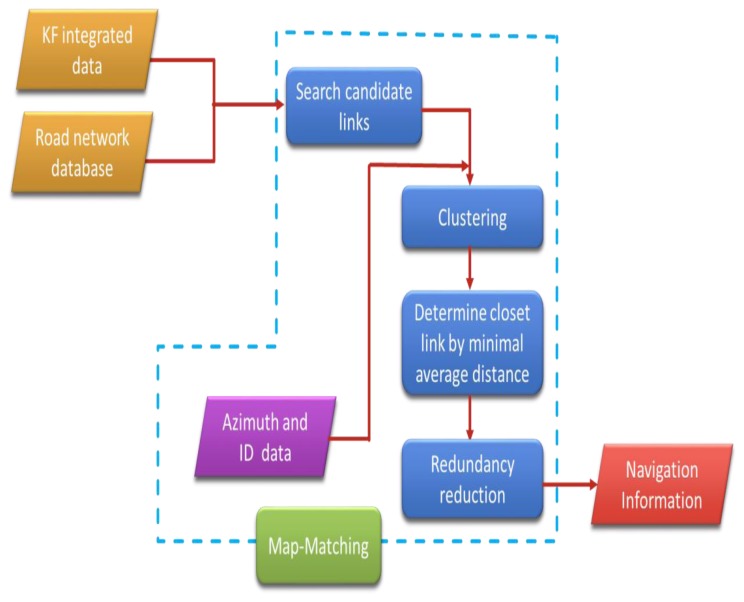
The map-matching algorithm.

**Figure 2.**
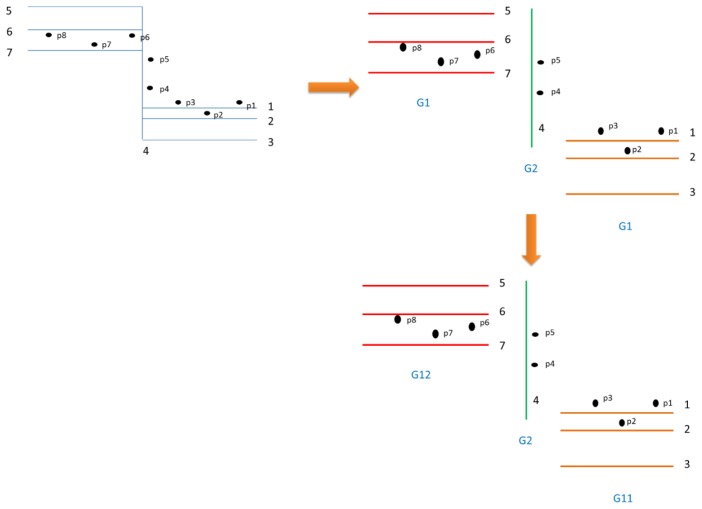
Two-step signal point clustering.

The purpose of developing the curve-to-curve algorithm was to reduce the position error drift during GPS outages. The method used in the study is modified from [8]. Figure 1 shows the processes in the algorithm. In the data processing, signal points are ordered in time and then point ID are attached. A link represents a road in the map and the road azimuth is known. The steps are described as follows:

1. Integrate GPS and IMU data using the KF: Extended KF (EKF) is used to integrate GPS and INS data based on a loosely coupled scheme. The details of EKF can be found in previous researches [3]. In this study, a linear approximation of a nonlinear function is proposed. The EKF can be simplified to the KF algorithm. The equations of the algorithm are shown in Equations (1)–(11).
2. Search candidate road links in a 30-m trajectory buffer and project points to links: The purpose of this step is to determine all link candidates and project signal points to candidate links for map matching. Firstly, the buffer is created based on the GPS/ INS trajectory. All links within the buffer will be defined as the candidate road links.
3. Cluster point sets considering projected link azimuth and point ID continuity: Signal points and candidate road links are stored in a database of a GIS. When the position errors in some points are large, the projected link azimuth and point ID are vital information to facilitate dividing links into point clusters. Figure 2 illustrates the clustering process. Point sets in each road link can be separated when considering projected link azimuth and point ID information. *G_i_* and *G_ij_* are defined as the clustering point sets based on link azimuth *i* and the ones based on link azimuth *i* and section *j*. A two-step clustering approach is shown. For example, the point sets are firstly separated into *G_2_* (p4, 5) and the other cluster *G_1_* (p1, 2, 3, 6, 7, 8) based on the projected link azimuth, and then are separated into *G_11_* (p1, 2, 3) and *G_12_* (p6, 7, 8) according to point ID continuity.
4. Determine the closest link in each point set: The objective is to determine the minimum distance from a point set to a link as the map matching indicator. If the minimum distance from a point set to a candidate link is determined, the link with the minimal distance is identified as the matching link:
  (12) $$Li=\text{arg}\;\text{min}(D_{ij})$$
  where *D_ij_* is the distance from a point set *i* to a link *j; Li* is the matching link in a point set *i*. For example, in Figure 2, the link with the minimal average distance is the best-matched link such as *L*1 link 1 in *G_11_*, *L*2: link 4 in *G_2_*, and *L*3: link 6 in *G_12_*. However, the point-to-curve algorithm skips step 4 and directly determines the closest link in each point.
5. Reduce redundancy: When the testing motorcycle stops, the redundancy is a large amount of duplicated random points. In the model, redundancy can be identified and removed automatically based on velocity information:
  (13) $$if\;v_{i}<v_{\text{thres}},\;\text{then pi is a redundant point}.$$
  where *v_i_* is velocity of the vehicle at point *pi*; *v_thres_* is the threshold velocity in motorcycle stopping.

## 3. Experiments and Materials

**Figure 3.**
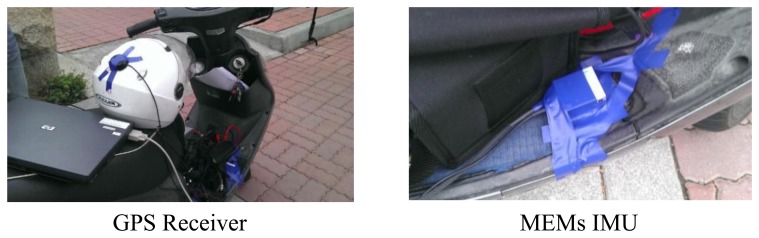
The GPS Receiver and MEMs IMU mounted on a motorcycle.

Tests were performed in Tainan City to assess the efficacy of both algorithms. The test trajectory length was approximately 6 km and the total experiment time was about 1,500 s. The MIDG II low-cost IMU and single frequency GPS receiver were mounted on a motorcycle to collect data (Figure 3). Table 1 shows the MIDG II specifications. The MIDG II is a GPS-aided INS for use in obtaining attitude, position, velocity, acceleration, and angular rates for navigation.

## 4. Results and Discussion

**Figure 4.**
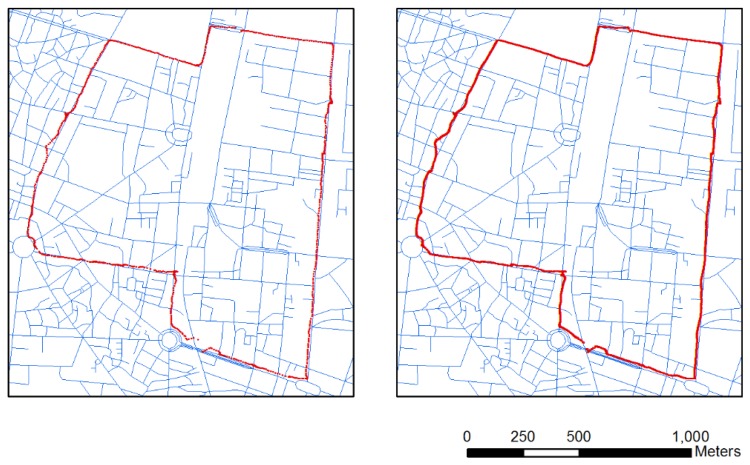
Raw GPS (**Left**) and integrated GPS/INS point data by KF (**Right**).

Figure 4 shows the raw GPS point data (Left panel) and GPS/INS data integrated by KF (Right panel). Although GPS signals are often unavailable due to signal outages in urban canyons, the MEMS-based inertial sensor technology has high-frequency signals without outages for low-cost integrated vehicle navigation systems [9]. An integrated GPS and INS will overcome the outages, but the KF cannot compensate all the drift errors caused by both sensors on the motorcycle platform. The resulting trajectory doesn't match well the road map used as the reference. The average error of each integrated point to a matching line is 5.09 m (right panel in Figure 4). Compared to the average errors of only GPS from each point to the matching road, an INS/GPS system with an average position error is still large because the platform is a motorcycle. Motorcycles are not as stable as vehicles. In the system, we don't consider the nonholonomic constraint (NHC) that limits the velocity of the vehicle in the plane perpendicular to the forward direction. Thus, map matching is used directly to overcome the problem

**Figure 5.**
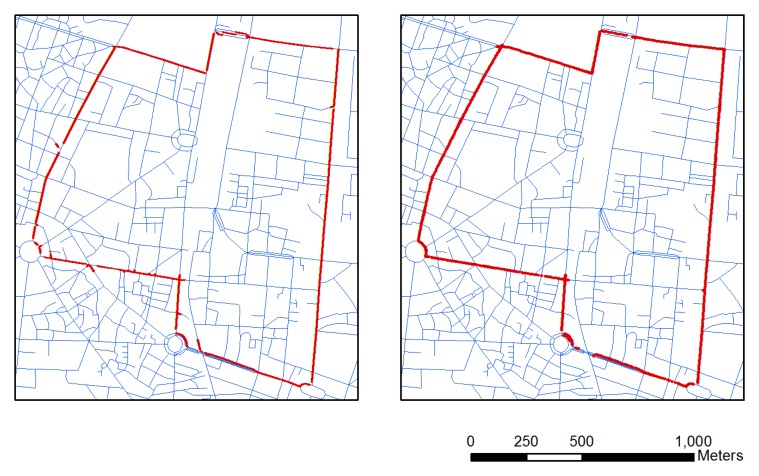
Point-to-curve (**Left**) and curve-to-curve (**Right**) map-matching results.

**Figure 6.**
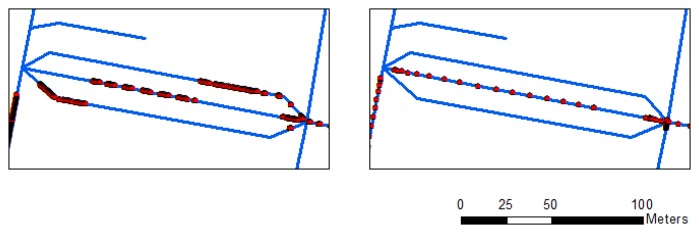
Details of point-to-curve (**Left**) and curve-to-curve (**Right**) map-matching results.

Figure 5 displays the point-to-curve (left) and curve-to-curve (right) map-matching results. Average errors for each point in both matching approaches are 1.73 and 0.61 m. The results show that the point-to-curve algorithm exhibits low accuracy, and some links are discontinuous when the motorcycle platform is not steady and the drift errors in the integrated data are large. The low-accuracy point-to-curve algorithm cannot identify all the right links for map matching (Left panel of Figure 5). However, the curve-to-curve algorithm mitigates most of the errors and improves the navigational solution (Right panel of Figure 5). Figure 6 shows the details of matching result comparisons of the two algorithms. The matched links are separated into three sub-links using the point-to-curve algorithm (Left panel of Figure 6), but can be improved using the curve-to-curve algorithm (Right panel of Figure 6).

**Figure 7.**
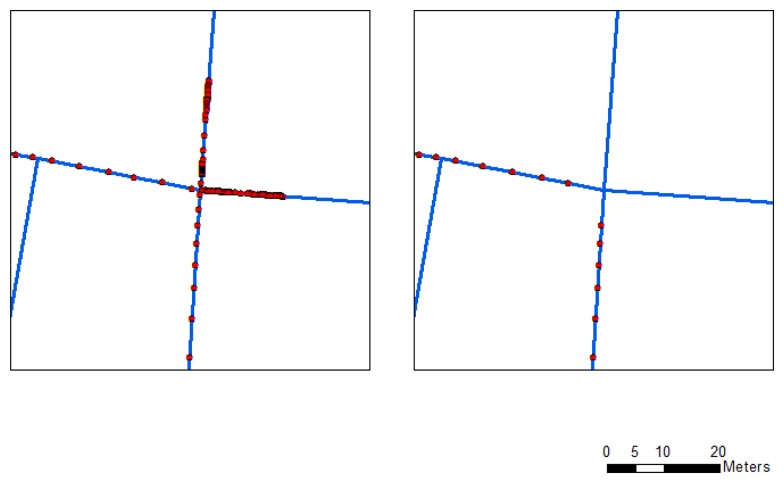
Map matching before (**Left**) and after signal redundancy reduction (**Right**).

Most of the limitations of existing algorithms have been explained in [7]. Two major limitations of map matching are incorrect matches at junctions or parallel roads (Equation (1)) and redundant points when the motorcycle stops (Equation (2)). The difficulty of identifying the correct link at a junction using the traditional map-matching procedures is observable in the left panel of Figure 6. The point-to-curve algorithm assigns an incorrect link because the algorithm does not take all azimuth (heading) information of the trajectory into account. In our approach, the heading information for each point set is considered in the map matching. This information implies that the trajectory is the same link. The algorithm accurately matches the correct track with the road map (Right panel of Figure 6). In the system, KF has not considered the constraint of zero velocity update, however, many redundant points occurred when the motorcycle stopped in waiting areas and crossroads. The problem of redundant points will be solved by using map matching. According to the model, redundancy can be removed using the curve-to-curve algorithm when the motorcycle stops (Right panel of Figure 7). The algorithm can overcome the redundant point problem (Left panel of Figure 7) by considering point ID continuity and road network information, therefore, the research develops a smart map matching algorithm for road networks. The algorithm can reduce the drift errors associated with the GPS outages, incorrect matches, and redundant points when the motorcycle is stationary for the precise identification of the correct link.

## 5. Conclusions

This study applies data fusion and a map-matching model in identifying the relationship between GPS/INS measured data and road map data for a robust navigation solution. The algorithm is suitable for integrating GPS and INS data into map matching in urban areas, and improves the incorrect routes based on the point-to-curve map matching. Therefore, even using a low-cost IMU or an unstable platform, the curve-to-curve map-matching algorithm can identify the right routes. Consequently, the proposed scheme can provide consistent navigation solutions with improved sustainability in GPS-denied environments. The best routes of map matching can be determined by reducing the irrational routes in GPS-denied environments. Future studies will be conducted to extend the procedure from 2-D to 3-D in land vehicular navigation systems. In addition, further study of personal mobile devices for real-time pedestrian navigation will be undertaken.

## Figures and Tables

**Table 1.** MIDG II specification.

**Physical Characteristics**	**IMU Performance**
Output rate (Hz)	50
Gyro bias (degree/h)	47
Gyro scale factor (ppm)	5,000
Accelerometer bias (mg)	6.0
Accelerometer scale factor (ppm)	19,700
